# Incidence of myeloid neoplasms in Spain (2002–2013): a population-based study of the Spanish network of cancer registries

**DOI:** 10.1038/s41598-021-03734-6

**Published:** 2022-01-10

**Authors:** Marta Solans, Arantza Sanvisens, Alberto Ameijide, Susana Merino, Dolores Rojas, Araceli Alemán, Emilia Banqueri, Matilde Chico, Ana Isabel Marcos, Visitación de Castro, Leire Gil, Arantza López de Munain, Montse Puigdemont, Maria-José Sánchez, Josefina Perucha, Patricia Ruiz-Armengol, Mª Dolores Chirlaque, Marcela Guevara, Marià Carulla, Rafael Marcos-Gragera

**Affiliations:** 1 Research Group on Statistics, Econometrics and Health (GRECS), University of Girona, Girona, Spain; 2 CIBER of Epidemiology and Public Health (CIBERESP), Madrid, Spain; 3 Epidemiology Unit and Girona Cancer Registry, Josep Carreras Leukaemia Research Institute, Girona, Spain; 4 Tarragona Cancer Registry, Cancer Prevention and Epidemiology Service, Sant Joan de Reus University Hospital, Tarragona, Spain; 5 Department of Health, Asturias Cancer Registry, Public Health Directorate, Asturias, Spain; 6 Canary Islands Cancer Registry, Public Health Directorate, Canary Islands Government, Las Palmas, Spain; 7 Castellón Cancer Registry, Public Health Directorate, Valencian Government, Castellón, Spain; 8 Ciudad Real Cancer Registry, Health and Social Welfare Authority, Castile-La Mancha, Spain; 9 Cuenca Cancer Registry, Health and Social Welfare Authority, Castile-La Mancha, Spain; 10 Basque Country Cancer Registry, Basque Government, Vitoria-Gasteiz, Spain; 11 Epidemiology Unit and Girona Cancer Registry, Oncology Coordination Plan, Catalan Institute of Oncology, Girona Biomedical Research Institute Dr. Josep Trueta (IDIBGI), Girona, Spain; 12 Granada Cancer Registry, Andalusian School of Public Health (EASP), Instituto de Investigación Biosanitaria Ibs.GRANADA, University of Granada, Granada, Spain; 13 La Rioja Cancer Registry, Epidemiology and Health Prevention Service, Logroño, Spain; 14 Mallorca Cancer Registry, Public Health and Participation Department, Palma de Mallorca, Spain; 15 Department of Epidemiology, Regional Health Authority, IMIB-Arrixaca, Murcia University, Murcia, Spain; 16 Navarra Cancer Registry, Navarra Public Health Institute, Navarra Institute for Health Research (IdiSNA), Pamplona, Spain

**Keywords:** Myelodysplastic syndrome, Myeloproliferative disease, Epidemiology

## Abstract

Comprehensive population-based data on myeloid neoplasms (MNs) are limited, mainly because some subtypes were not recognized as hematological cancers prior to the WHO publication in 2001, and others are too rare to allow robust estimates within regional studies. Herein, we provide incidence data of the whole spectrum of MNs in Spain during 2002–2013 using harmonized data from 13 population-based cancer registries. Cases (*n* = 17,522) were grouped following the HAEMACARE groupings and 2013-European standardized incidence rates (ASR_E_), incidence trends, and estimates for 2021 were calculated. ASR_E_ per 100,000 inhabitants was 5.14 (95% CI: 5.00–5.27) for myeloproliferative neoplasms (MPN), 4.71 (95% CI: 4.59–4.84) for myelodysplastic syndromes (MDS), 3.91 (95% CI: 3.79–4.02) for acute myeloid leukemia, 0.83 (95% CI: 0.78–0.88) for MDS/MPN, 0.35 (95% CI: 0.32–0.39) for acute leukemia of ambiguous lineage, and 0.58 (95% CI: 0.53–0.62) for not-otherwise specified (NOS) cases. This study highlights some useful points for public health authorities, such as the remarkable variability in incidence rates among Spanish provinces, the increasing incidence of MPN, MDS, and MDS/MPN during the period of study, in contrast to a drop in NOS cases, and the number of cases expected in 2021 based on these data (8446 new MNs).

## Introduction

Hematological malignancies are the fourth most frequently diagnosed group of cancers worldwide^1^, with an annual incidence rate of 39.37 per 100,000 inhabitants in Europe in 2000–2002^2^. They encompass a heterogeneous group of diseases with diverse etiology, presentation, and outcomes. Our understanding of these neoplasms has evolved rapidly during the recent decades, resulting in multiple classification updates. Currently, the World Health Organization (WHO) classification of hematologic malignancies, first published in 2001^3^ and later updated in 2008^4^ and 2016^5^, is the gold standard for the study of these neoplasms. Such continuous definition refinements, however, have posed significant problems for population-based cancer registries to present complete and accurate data for the full spectrum of hematological neoplasms^6^, and particularly for myeloid neoplasms (MNs).

MNs are a group of clonal disorders characterized by excessive proliferation, impaired self-renewal and/or altered differentiation of hematopoietic stem cells and myeloid progenitor cells. Broadly, MNs are classified into four large categories: acute myeloid leukemia (AML), myeloproliferative neoplasms (MPN), myelodysplastic syndromes (MDS), and MDS/MPN overlap syndromes. However, MDS and several MPN subtypes were first recognized as malignant disorders—and thus, reportable to cancer registries—in 2000, with the publication of the third revision of the International Classification of Disease for Oncology (ICD-O-3)^7^. Regarding AML, past epidemiological studies were prone to group all leukemias together, at best differencing by age range (i.e. pediatric/adult) and chronicity (i.e. acute/chronic), but ignoring cell lineage differences (i.e. lymphoid/myeloid). This situation changed after correspondence was established between the WHO classifications and the ICD-O-3 codes. Since then, several studies using the largest European^2,8^ and North American^9–12^ datasets, as well as from hematology-specialized cancer registries^13,14^ have reported detailed epidemiological data of MNs. Still, and given that most entities are too rare to make power robust estimates within regional studies, population-based data of several clinically meaningful histological subtypes are limited. Particularly in Spain, there are no available comprehensive nationwide estimates, with only one previous study providing regional data^15^.

The aim of this study was to assess the incidence of MNs subtypes in Spain over the period 2002–2013, and to estimate the number of MNs expected in 2021, using harmonized data from the Spanish Network of Cancer Registries (REDECAN).

## Methods

REDECAN was created in 2010 in order to develop standards for cancer registration, undertake quality audits, and promote the use of cancer surveillance data within Spain^16,17^. Currently, it comprises 15 population-based cancer registries, 14 of them covering the entire population and one of them only children. In particular, 13 population based-cancer registries (i.e. Asturias, Canary Islands, Castellón, Ciudad Real, Cuenca, Euskadi, Girona, Granada, La Rioja, Mallorca, Murcia, Navarra, and Tarragona) contributed to this study (Table 1), covering ∼26% of the total Spanish population in January 2013 (12,143,157 out of 47,129,783)^18^.

**Table 1:** Period of study, number of cases of myeloid neoplasms, and quality indicators of data provided by Spanish provinces.

Cancer registry	Period	*N* cases	Person-years	Quality indicators		
				MV (%)	NOS^1^ (%)	DCOs (%)
Asturias	2002–2010	1238	9,603,522	95.0	3.9	0.0
Canary Islands						
Gran Canaria	2002–2013	975	12,159,144	96.9	3.1	1.7
Tenerife	2002–2013	1018	11,122,121	96.2	4.3	3.0
Castellón	2004–2013	763	5,721,562	99.2	2.5	0.8
Ciudad Real	2004–2011	527	4,087,571	98.9	5.1	0.9
Cuenca	2002–2011	452	2,093,316	93.8	6.4	3.3
Euskadi						
Álava	2002–2013	649	3,683,786	97.5	2.9	0.8
Gipuzkoa	2002–2013	1308	8,330,687	92.6	9.9	1.4
Bizkaia	2002–2013	2380	13,707,586	97.9	2.8	1.7
Girona	2002–2013	1573	8,233,631	96.6	1.3	2.9
Granada	2002–2013	1105	10,598,249	96.1	2.2	3.9
La Rioja	2002–2013	612	3,678,906	96.7	1.5	2.0
Mallorca	2003–2012	1180	10,101,043	97.5	2.7	2.1
Murcia	2002–2010	1552	12,091,278	94.3	4.3	1.2
Navarra	2002–2012	842	6,635,158	95.6	1.4	1.5
Tarragona	2002–2013	1308	8,854,188	96.7	4.0	3.0
**Spain**	2002–2013	17,522	130,701,748	96.3	3.6	1.9

^1^NOS cases included the following ICD-O-3 codes: 9860, 9800.

*MV* microscopically verified, *NOS* not-otherwise specified, *DCO* death certificate only.

All incident MNs registered from 2002 to 2013 (or the available period) were included in the analyses. Cases were codified using the ICD-O-3^7,19^, and classified following the HAEMACARE scheme^2^, a European project aimed to improve standardization of epidemiological information on hematological malignancies (Table 2). In brief, MNs were grouped into six broad categories: MPN, MDS/MPN, MDS, AML, acute leukemia with ambiguous lineage, and not otherwise specified (NOS). Within AML, the following groupings were considered: AML with recurrent cytogenetic abnormalities, AML with multilineage dysplasia, AML and MDS therapy-related, AML not otherwise categorized (NOC), and AML NOS. In the 2001 WHO classification^3^, the number of blast cells to define AML decreased from 30 to 20% so that some conditions previously considered MDS, such as refractory anemia with excess blasts in transformation (9984/3), were included with the AMLs. In line with previous studies^20^, we grouped these cases as AML with multilineage dysplasia (9895/3) as this cytological property is characteristic of MDS. Likewise, therapy related-MDS (9987/3) were included in the AML therapy related (9920/3) subgroup, and cases of chronic myeloid leukemia NOS (CML NOS) (9863/3) were grouped with CML BCR-ABL positive (9875/3). Finally, in cases of hematological transformation, only the first tumor was considered for incidence^21^.

**Table 2:** List of ICD-O-3 codes included in the analysis, number of cases, median age and crude and age-standardized incidence rates of myeloid neoplasms diagnosed during 2002–2013 in Spain.

	ICD-O-3 codes	*N*	Median age (IQR)	CR total	ASR_E_ total	ASR_E_ men	ASR_E_ women	Sex ratio
**MPN**		5872	69 (54–78)	4.68	5.14 (5.00; 5.27)	6.08 (5.86; 6.3)	4.41 (4.24; 4.58)	1.38
Chronic myeloid leukemia	9863, 9875	1324	62 (46–76)	1.06	1.13 (1.06; 1.19)	1.45 (1.35; 1.56)	0.85 (0.78; 0.92)	1.71
Polycythemia vera	9950	1064	71 (59–78)	0.85	0.95 (0.89; 1.01)	1.12 (1.02; 1.21)	0.82 (0.75; 0.89)	1.37
Primary myelofibrosis	9961	457	71 (63–77)	0.36	0.41 (0.38; 0.45)	0.65 (0.58; 0.72)	0.23 (0.19; 0.27)	2.83
Essential thrombocythemia	9962	2401	68 (53–78)	1.91	2.10 (2.01; 2.18)	2.14 (2.01; 2.27)	2.09 (1.98; 2.20)	1.02
Chronic neutrophilic/eosinophilic leukemia	9963–9964	53	66 (55–74)	0.04	0.05 (0.03; 0.06)	0.07 (0.04; 0.09)	0.03 (0.02; 0.04)	2.33
Mastocytosis	9740–9742	83	54 (36–66)	0.07	0.07 (0.05; 0.09)	0.08 (0.06; 0.10)	0.06 (0.04; 0.08)	1.33
MPN unclassifiable	9960	490	75 (63–81)	0.39	0.43 (0.39; 0.47)	0.58 (0.51; 0.65)	0.33 (0.29; 0.38)	1.76
**MDS/MPN**		912	77 (70–83)	0.73	0.83 (0.78; 0.88)	1.35 (1.24; 1.46)	0.47 (0.42; 0.53)	2.87
Chronic myelomonocytic leukemia	9945	763	77 (70–83)	0.61	0.70 (0.65; 0.75)	1.16 (1.06; 1.26)	0.38 (0.33; 0.43)	3.05
Juvenile myelomonocytic leukemia	9846	8	0 (0–0)	0.01	0.01 (0.00; 0.01)	0.01 (0.00; 0.01)	0.00 (0.00; 0.01)	-
Atypical chronic myeloid leukemia	9876	49	73 (59–80)	0.04	0.04 (0.03; 0.06)	0.07 (0.05; 0.09)	0.02 (0.01; 0.04)	3.50
MDS/MPN, unclassifiable	9975	92	81 (73–86)	0.07	0.08 (0.07; 0.1)	0.11 (0.08; 0.14)	0.07 (0.05; 0.09)	1.57
**MDS**		5213	78 (71–83)	4.16	4.71 (4.59; 4.84)	6.54 (6.3; 6.79)	3.47 (3.33; 3.62)	1.88
MDS with single lineage dysplasia	9980, 9991	731	78 (72–83)	0.58	0.66 (0.61; 0.71)	0.87 (0.78; 0.95)	0.52 (0.46; 0.57)	1.67
MDS with ring sideroblasts and single lineage dysplasia	9982	471	77 (70–82)	0.38	0.42 (0.39; 0.46)	0.62 (0.54; 0.69)	0.29 (0.25; 0.33)	2.14
MDS with excess of blasts	9983	912	75 (68–80)	0.73	0.82 (0.77; 0.88)	1.19 (1.09; 1.29)	0.54 (0.48; 0.60)	2.20
MDS with multilineage dysplasia	9985	782	77 (70–82)	0.62	0.70 (0.65; 0.75)	1.02 (0.92; 1.11)	0.47 (0.42; 0.53)	2.17
MDS associated with isolated del(5q)	9986	116	75 (67–79)	0.09	0.10 (0.09; 0.12)	0.06 (0.04; 0.08)	0.14 (0.11; 0.17)	0.43
MDS unclassifiable	9989	2201	80 (73–86)	1.76	2.01 (1.92; 2.09)	2.79 (2.63; 2.96)	1.51 (1.42; 1.61)	1.85
**AML**		4498	68 (50–78)	3.59	3.91 (3.79; 4.02)	4.75 (4.55; 4.94)	3.28 (3.14; 3.42)	1.45
AML with recurrent cytogenetic abnormalities		608	49 (35–67)	0.48	0.5 (0.46; 0.54)	0.55 (0.49; 0.61)	0.46 (0.41; 0.52)	1.20
AML with t(8;21)(q22;q22)	9896	115	60 (40–73)	0.09	0.10 (0.08; 0.12)	0.11 (0.08; 0.14)	0.09 (0.06; 0.11)	1.22
AML with 11q23 abnormalities	9897	17	67 (56–77)	0.01	0.01 (0.01; 0.02)	0.02 (0.01; 0.03)	0.01 (0.00; 0.02)	2.00
AML with inv(16)(p13;q22) or t(16;16)(p13;q11)	9871	45	51 (29–72)	0.04	0.04 (0.03; 0.05)	0.06 (0.04; 0.08)	0.02 (0.01; 0.03)	3.00
AML with t(15;17)(q22;q11-12)	9866	431	47 (33–63)	0.34	0.35 (0.32; 0.38)	0.37 (0.32; 0.42)	0.34 (0.30; 0.39)	1.09
AML with multilineage dysplasia	9895, 9984	494	74 (66–80)	0.39	0.44 (0.40; 0.48)	0.61 (0.54; 0.68)	0.32 (0.27; 0.36)	1.91
AML and MDS therapy related	9920, 9987	120	66 (56–73)	0.10	0.11 (0.09; 0.13)	0.10 (0.07; 0.13)	0.12 (0.09; 0.15)	0.83
AML NOC		2136	66 (49–77)	1.70	1.85 (1.77; 1.93)	2.26 (2.13; 2.40)	1.53 (1.43; 1.62)	1.48
AML minimal differentiated	9872	334	72 (57–79)	0.27	0.29 (0.26; 0.33)	0.39 (0.33; 0.45)	0.23 (0.19; 0.27)	1.70
AML without maturation	9873	431	66 (49–76)	0.34	0.37 (0.34; 0.41)	0.44 (0.38; 0.49)	0.32 (0.27; 0.36)	1.38
AML with maturation	9874	324	61 (46–73)	0.26	0.28 (0.25; 0.31)	0.30 (0.25; 0.34)	0.26 (0.22; 0.30)	1.15
Acute myelomonocytic leukemia	9867	355	66 (46–77)	0.28	0.31 (0.27; 0.34)	0.38 (0.33; 0.44)	0.24 (0.21; 0.28)	1.58
Acute monoblastic and monocytic leukemia	9891	446	65 (46–76)	0.36	0.38 (0.35; 0.42)	0.48 (0.42; 0.54)	0.31 (0.27; 0.36)	1.55
Acute erythroid leukemia	9840	137	70 (56–78)	0.11	0.12 (0.10; 0.14)	0.17 (0.13; 0.20)	0.08 (0.06; 0.10)	2.13
Acute megakaryoblastic leukemia	9910	30	48 (2–67)	0.02	0.02 (0.02; 0.03)	0.03 (0.01; 0.04)	0.02 (0.01; 0.03)	1.50
Acute basophilic leukemia	9870	2	80 (77–83)	0.00	0.00 (0.00; 0.00)	0.00 (0.00; 0.01)	0.00 (0.00; 0.00)	-
Acute panmyelosis with myelofibrosis	9931	43	71 (64–76)	0.03	0.04 (0.03; 0.05)	0.05 (0.03; 0.07)	0.03 (0.02; 0.05)	1.67
Myeloid sarcoma	9930	34	66 (41–76)	0.03	0.03 (0.02; 0.04)	0.03 (0.02; 0.05)	0.02 (0.01; 0.04)	1.50
AML NOS	9861	1140	73 (58–80)	0.91	1.01 (0.95; 1.07)	1.23 (1.13; 1.33)	0.85 (0.78; 0.93)	1.45
**Acute leukemia of ambiguous lineage**		395	75 (60–83)	0.32	0.35 (0.32; 0.39)	0.41 (0.35; 0.47)	0.30 (0.26; 0.35)	1.37
Acute leukemia, NOS	9801	346	76 (64–83)	0.28	0.31 (0.28; 0.34)	0.35 (0.30; 0.41)	0.28 (0.23; 0.32)	1.25
Biphenotypic acute leukemia	9805, 9807–9809	49	57 (37–72)	0.04	0.04 (0.03; 0.05)	0.06 (0.04; 0.08)	0.03 (0.01; 0.04)	2.00
**Unknown myeloid neoplasms**	9860, 9800, 9965, 9967	632	81 (73–86)	0.50	0.58 (0.53; 0.62)	0.79 (0.7; 0.88)	0.45 (0.40; 0.50)	1.76
**Total cases**		17,522	73 (60–81)	13.97	15.52 (15.29; 15.75)	19.92 (19.51; 20.33)	12.39 (12.12; 12.67)	1.61

*IQR* interquartile range, *CR* crude rate, *ASR*_*E*_ Age-standardized rate (2013 European population), *AML* acute myeloid leukemia, *MPN* myeloproliferative neoplasms, *MDS* myelodysplastic syndromes, *MN* myeloid neoplasms, *NOC* not otherwise categorized, *NOS* not otherwise specified. Rates are expressed per 100,000 person-years.

Crude rate (CR) and age-standardized incidence rate using the 2013 European standard population (ASR_E_) were calculated using population data provided by the National Statistics Institute (Instituto Nacional de Estadística—INE)^18^, and expressed per 100,000 person-years. Poisson regression models were used to analyze the overall incidence time trends and to estimate the annual percent change (APC). The number of cases in Spain for 2021 was determined by applying to the 2021 Spanish population^18^ the age-specific rates estimated for the year 2021. The latter were obtained by applying the APC (period 2002–2013) to the last quinquennium of known incidence (i.e. 2009–2013). All analyses were performed using R software (version 3.6.1)^22^.

### Ethics approval

This study is based on data from cancer registries gathered in the Spanish network of cancer registries (REDECAN). The public health administration of each autonomous community/province* authorized the collection and use of this data for its analysis without requirement of informed consent and ethical approval, covered by the Spanish general and public health laws 14/1986 and 33/2011.

*The authorizing bodies for each autonomous community/province are listed below: Asturias: Sección de Información Sanitaria. Servicio de Evaluación de la Salud, Calidad y Programas de la Dirección General de Salud Pública. Consejería de Sanidad; Canary Islands: Servicio de Epidemiología y Prevención. Dirección General de Salud Pública. Servicio Canario de la Salud; Castellón: Conselleria de Sanitat. Dirección General de Salud Pública; Ciudad Real: Consejería de Sanidad y Asuntos Sociales de la Junta de Comunidades de Castilla-La Mancha; Cuenca: Consejería de Sanidad y Asuntos Sociales. Junta de Comunidades de Castilla la Mancha; Euskadi: Dirección de Planificación, Ordenación y Evaluación Sanitarias. Departamento de Salud. Gobierno Vasco; Girona: Plan Director de Oncología-Instituto Catalán de Oncología; Granada: Consejería de Salud de la Junta de Andalucía, adscrito para su desarrollo a la Escuela Andaluza de Salud Pública (EASP); La Rioja: Servicio de Epidemiología y Prevención Sanitaria de la Consejería de Salud y Servicios Sociales del Gobierno de La Rioja; Mallorca: Dirección General de Salud Pública y Participación; Murcia: Consejería de Salud de Murcia; Navarra: Departamento de Salud del Gobierno de Navarra. Instituto de Salud Pública y Laboral de Navarra; Tarragona: Hospital Universitario Sant Joan de Reus.

## Results

MNs accounted for 30.81% (*n* = 17,522) of all hematologic malignancies (*n* = 56,777) diagnosed in Spanish population covered by cancer registries between 2002 and 2013. The quality and completeness of each registry, together with the study period and the total cases are detailed in Table 1. Of the total, 96.3% of the cases had microscopic confirmation, 3.6% were NOS cases, and 1.9% were recorded exclusively by death certificate (DCO). In particular, 33.5% of cases were MPN, 29.8% MDS, 25.7% AML, 5.2% MDS/MPN, 2.3% acute leukemia of ambiguous lineage, and the remaining 3.6% were NOS cases.

**Figure 1:**
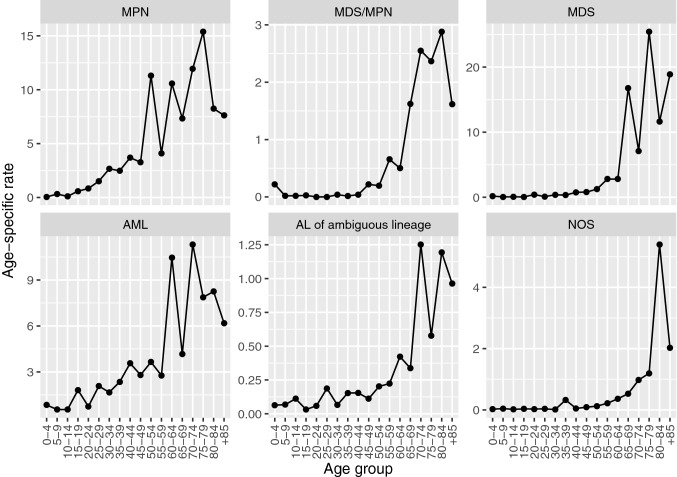
Age-specific incidence rates of broad categories of myeloid neoplasms diagnosed in Spain during 2002–2013. *AML* acute myeloid leukemia, *AL* acute leukemia, *MDS* myelodysplastic syndromes, *MPN* myeloproliferative neoplasms, *NOS* not-otherwise specified. Rates are expressed per 100,000 person-years.

**Figure 2:**
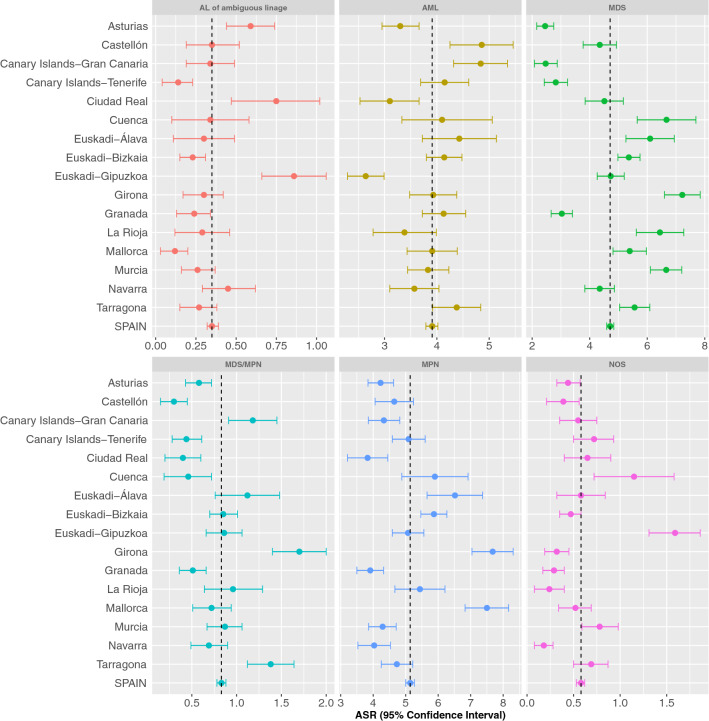
Age-standardized incidence rates (ASR_E_) of myeloid neoplasms diagnosed in Spain during 2002–2013 by region. *AML* acute myeloid leukemia, *AL* acute leukemia of ambiguous lineage, *MDS* myelodysplastic syndromes, *MPN* myeloproliferative neoplasms, *NOS* not-otherwise specified. Rates are expressed per 100,000 person-years.

Table 2 shows the number of cases, median age and incidence rates of all MNs according to histological subtype. The overall CR was 13.97 (95% CI 13.77; 14.18), and the overall ASR_E_ was 15.52 (95% CI: 15.29; 15.75), being 19.92 (95% CI 19.51; 20.33) in men and 12.39 (95% CI 12.12; 12.67) in women. There was a marked male predominance (9,650 cases in men (55.1%), sex ratio = 1.61), and the median age at diagnosis was 73 years (interquatile range (IQR) 60–81 years). Moreover, incidence increased markedly with age, reaching a maximum around 75–79 years in most subgroups (Fig. 1). ASR_E_ of MNs by cancer registry are displayed in Fig. 2. There were significant differences across different cancer registries (especially regarding MDS, MPN, and MDS/MPN), with the highest overall rates in Girona (21.14, 95% CI 20.09; 22.19), and the lowest rates observed in Asturias (11.61, 95% CI 10.95; 12.26) and Granada (12.11, 95% CI 11.38; 12.83).

### Myeloproliferative neoplasms

The CR and ASR_E_ for MPN were 4.68 (95% CI 4.56; 4.80) and 5.14 (95% CI 5.00; 5.27), respectively. The most frequent subtype was essential thrombocythemia (41% of cases, ASR_E_ = 2.10 (95% CI 2.01; 2.18)), followed by chronic myeloid leukemia (23% of cases, ASR_E_ = 1.13 (95% CI 1.06; 1.19)), and polycythemia vera (18%, ASR_E_ = 0.95 (95% CI 0.89; 1.01)) (Table 2). Median age (IQR) at diagnosis was 69 (54–78) years, being slightly lower in cases with mastocytosis, 54 (36–66) years. Higher rates in men than in women were reported in almost all subtypes, being particularly higher in primary myelofibrosis (sex ratio = 2.83), while no difference by sex was seen in essential thrombocythemia (sex ratio = 1.02). The incidence of MPN increased across the period in 2002–2013, with an APC of 1.6% (95% CI 0.8; 2.4) (Table 3). Moreover, results by specific subtype indicate that this increment in the incidence was namely due to the contribution of essential thrombocythemia (4.6%, 95% CI 3.3; 5.9), primary myelofibrosis (3.5%, 95% CI 0.6; 6.5), and polycythemia vera (2.2%, 95% CI 0.3; 4.1). Conversely, we evidenced a decrease in the incidence of chronic myeloid leukemia (− 2.1%, 95% CI − 3.7; − 0.4) and MPN NOS (− 5.0%, 95% CI − 7.6; − 2.4).

**Table 3:** Incidence trends of myeloid neoplasms diagnosed during 2002–2013 in Spain.

	*N*	APC (95% CI)^1^	
**MPN**	5872	1.6 (0.8; 2.4)	*
Chronic myeloid leukemia	1324	− 2.1 (− 3.7; − 0.4)	*
Polycythemia vera	1064	2.2 (0.3; 4.1)	*
Primary myelofibrosis	457	3.5 (0.6; 6.5)	*
Essential thrombocythemia	2401	4.6 (3.3; 5.9)	*
Chronic neutrophilic/eosinophilic leukemia	53	− 1.1 (− 9.0; 7.5)	
Mastocytosis	83	0.6 (− 5.9; 7.5)	
MPN unclassifiable	490	− 5.0 (− 7.6; − 2.4)	*
**MDS/MPN**	912	6.9 (4.8; 9.1)	*
Chronic myelomonocytic leukemia	763	5.3 (3.0; 7.6)	*
Juvenile myelomonocytic leukemia	8	–	
Atypical chronic myeloid leukemia	49	10.1 (0.8; 20.4)	*
MDS/MPN, unclassifiable	92	19.7 (11.6; 28.1)	*
**MDS**	5213	1.3 (0.4; 2.1)	*
MDS with single lineage dysplasia	731	− 11.9 (− 13.9; − 9.8)	*
MDS with ring sideroblasts and single lineage dysplasia	471	− 2.5 (− 5.2; 0.3)	
MDS with excess of blasts	912	0.8 (− 1.2; 2.8)	
MDS with multilineage dysplasia	782	19.1 (16.3; 21.9)	*
MDS associated with isolated del(5q)	116	13.7 (7.2; 20.6)	*
MDS unclassifiable	2201	0.8 (− 0.5; 2.1)	
**AML**	4498	0.8 (− 0.5; 2.1)	
AML with recurrent cytogenetic abnormalities	608	4.2 (1.6; 6.7)	*
AML with multilineage dysplasia	494	4.6 (1.8; 7.5)	*
AML and MDS therapy related	120	22.3 (15.0; 30.0)	*
AML NOC	2136	1.4 (0.1; 2.8)	*
AML NOS	1140	− 5.2 (− 6.9; − 3.4)	*
**Acute leukemia of ambiguous lineage**	395	− 9.2 (− 12.0; − 6.3)	*
**Unknown myeloid neoplasms**	632	− 14.3 (− 16.5; − 12.1)	*
**Total cases**	**17,522**	0.7 (0.3; 1.2)	*

*p-value < 0.05.

*APC* annual percent change, *CI* confidence interval, *AML* acute myeloid leukemia, *MPN* myeloproliferative neoplasms, *MDS* myelodysplastic syndromes, *MN* myeloid neoplasms, *NOC* not-otherwise categorized, *NOS* not-otherwise specified.

### Myelodysplastic/myeloproliferative neoplasms

The CR and ASR_E_ for MDS/MPN were 0.73 (95% CI 0.68; 0.77) and 0.83 (95% CI 0.78; 0.88), respectively. By far, the most common subtype was chronic myelomonocytic leukemia (84% of cases), with an ASR_E_ of 0.70 (95% CI 0.65; 0.75) and a marked male predominance (sex ratio = 3.1) (Table 2). The median (IQR) age at diagnosis was 77 (70–83) years. The incidence of MDS/MPN rose markedly throughout 2002–2013, with an APC of 6.9% (95% CI: 4.8; 9.1) (Table 3).

### Myelodysplastic syndromes

The CR and ASR_E_ for MDS were 4.16 (95% CI 4.04; 4.27) and 4.71 (95% CI 4.59; 4.84), respectively (Table 2). The most frequent subtype was MDS with excess of blasts (17%, ASR_E_ = 0.82 (95% CI 0.77; 0.88)), closely followed by MDS with multilineage dysplasia (15%, ASR_E_ = 0.70 (95% CI 0.65; 0.75)) and MDS with single lineage dysplasia (14%, ASR_E_ = 0.66 (95% CI: 0.61; 0.71)], while 42% of the cases were MDS unclassifiable. Median age (IQR) at diagnosis was 78 (71–83) years, and incidence rates were higher in men, except in MDS associated with isolated del(5q), in which we noted a reverse sex ratio (0.43). There was a positive incidence trend of overall MDS throughout the period (APC = 1.3%, 95% CI 0.4; 2.1), mostly due to MDS with multilineage dysplasia (APC = 19.1%, 95% CI 16.3; 21.9) and MDS associated with isolated del(5q) (APC = 13.7%, 95% CI 7.2; 20.6). Conversely, cases of MDS with single lineage dysplasia decreased across the period of study (APC =  − 11.9%, − 13.9; − 9.8) (Table 3).

### Acute myeloid leukemia

The CR and ASR_E_ for AML were 3.59 (95% CI 3.48; 3.69) and 3.91 (95% CI 3.79; 4.02), respectively (Table 2). Among the 4498 AML cases, 47% were AML NOC, 14% were AML with recurrent cytogenetic abnormalities, 11% were AML with multilineage dysplasia, 3% were therapy-related, and 25% were NOS. Within AML with recurrent cytogenetic abnormalities (*n* = 608), the most frequent subtype was AML with t(15;17), followed by AML with t(8;21). A male predominance was shared across all subgroups (overall sex ratio = 1.45), with the exception of AML and MDS therapy related (sex ratio = 0.83). The median (IQR) age of AML patients was 68 (50–78) years, being lower in AML with cytogenetic abnormalities (49 (35–67) years). The incidence of overall AML was relatively stable over time, yet there was an increase in the incidence of specified cases in detriment of AML NOS cases (APC =  − 5.2%, 95% CI − 6.9; − 3.4) (Table 3).

### Acute myeloid leukemia with ambiguous lineage and NOS cases

There were 395 cases of acute myeloid leukemia with ambiguous lineage, 12% of them being biphenotypic acute leukemia. The overall CR and ASR_E_ were 0.32 (95% CI 0.28; 0.35) and 0.35 (95% CI 0.32; 0.39), respectively. Finally, there were 632 NOS cases (3.6% of the overall dataset), with a higher median age than that of the MNs as a whole [81 (73–86) years]. We evidenced a marked negative incidence trend across the period of study, both for acute leukemia of ambiguous lineage (APC =  − 9.2. 95% CI − 12.0; − 6.3), and NOS cases (APC =  − 14.3%, 95% − 16.5; − 12.1) (Table 3).

### Projections for 2021

Predicted incidence of MNs for 2021, overall and by sex, are detailed in Table 4. According to our projections, 8446 new incident cases of MNs will be diagnosed in Spain in 2021, of which 2835 will be MPN, 650 MSD/MPN, 2670 MDS, 2060 AML, and 106 acute leukemia of ambiguous lineage, and 126 NOS cases.

**Table 4:** Estimation of the incidence of myeloid neoplasms in Spain for 2021.

Subtype	Total			Men			Women		
	*N*	CR	ASR_E_	*N*	CR	ASR_E_	*N*	CR	ASR_E_
**MPN**	2835	5.99	5.70	1442	6.22	6.50	1393	5.77	4.91
Chronic myeloid leukemia	424	0.90	0.87	250	1.08	1.13	174	0.72	0.61
Polycythaemia vera	562	1.19	1.14	302	1.30	1.36	260	1.08	0.92
Primary myelofibrosis	251	0.53	0.53	187	0.81	0.84	64	0.27	0.23
Essential thrombocythemia	1421	3.00	2.81	622	2.68	2.80	799	3.31	2.81
Chronic neutrophilic/eosinophilic leukemia	18	0.04	0.04	14	0.06	0.07	3	0.01	0.01
Mastocytosis	30	0.06	0.06	11	0.05	0.05	20	0.08	0.07
MPN unclassifiable	129	0.27	0.26	56	0.24	0.25	73	0.30	0.26
**MDS/MPN**	**650**	**1.37**	**1.36**	**438**	**1.89**	**1.97**	**212**	**0.88**	**0.75**
Chronic myelomonocytic leukemia	522	1.10	1.10	365	1.57	1.65	157	0.65	0.55
Juvenile myelomonocytic leukemia	6	0.01	0.01	4	0.02	0.02	2	0.01	0.01
Atypical chronic myeloid leukemia	36	0.08	0.08	25	0.11	0.11	11	0.04	0.04
MDS/MPN unclassifiable	87	0.18	0.17	44	0.19	0.20	43	0.18	0.15
**MDS**	**2670**	**5.64**	**5.49**	**1591**	**6.86**	**7.17**	**1079**	**4.47**	**3.80**
MDS with single lineage dysplasia	162	0.34	0.33	95	0.41	0.43	67	0.28	0.24
MDS with ring sideroblasts and single lineage dysplasia	163	0.34	0.33	90	0.39	0.40	73	0.30	0.26
MDS with excess of blasts	432	0.91	0.91	301	1.30	1.36	132	0.55	0.46
MDS with multilineage dysplasia	677	1.43	1.42	453	1.95	2.04	224	0.93	0.79
MDS associated with isolated del(5q)	79	0.17	0.15	19	0.08	0.09	59	0.25	0.21
MDS unclassifiable	1157	2.44	2.35	633	2.73	2.85	524	2.17	1.84
**AML**	**2060**	**4.35**	**4.17**	**1098**	**4.74**	**4.95**	**961**	**3.98**	**3.39**
AML with recurrent cytogenetic abnormalities	338	0.71	0.68	169	0.73	0.76	169	0.70	0.60
AML with multilineage dysplasia	297	0.63	0.60	159	0.69	0.72	138	0.57	0.48
AML and MDS therapy related	101	0.21	0.20	42	0.18	0.19	59	0.24	0.21
AML NOC	977	2.06	1.99	556	2.40	2.50	422	1.75	1.49
AML NOS	346	0.73	0.69	173	0.74	0.78	173	0.72	0.61
**Acute leukemia of ambiguous lineage**	**106**	**0.22**	**0.21**	**48**	**0.21**	**0.22**	**58**	**0.24**	**0.20**
**Unknown myeloid neoplasms**	**126**	**0.27**	**0.26**	**72**	**0.31**	**0.33**	**54**	**0.22**	**0.19**
**Total cases**	8446	17.84	17.19	4689	20.22	21.14	3757	15.56	13.23

*CR* crude rate, *ASR*_*E*_ age-standardized rate (2013 European population), *AML* acute myeloid leukemia, *MPN* myeloproliferative neoplasms, *MDS* myelodysplastic syndromes, *MN* myeloid neoplasms, *NOC* not-otherwise categorized, *NOS* not-otherwise specified. Rates are expressed in 100,000 person-years.

## Discussion

Limited epidemiological data are available on the whole spectrum of hematologic disorders of the myeloid lineage. Within Europe, the most relevant data comes from two large European datasets, the RARECARE^8^ (1995–2002, *n* = 69,212) and HAEMACARE^2^ (2000–2002, *n* = 21,796) collaborative projects, and from two hemato-specialized registries, the Coté d’Or (France)^13^ (1980–2004, *n* = 5,086) and the UK Haematological Malignancy Research Network (HMRN)^14^ (2004–2015, *n* = 5,231). Our large-population-based study, which includes 17,522 cases diagnosed after the introduction of the WHO classification breakthrough, further complements these data by providing complete estimates of MNs burden in Spain during 2002–2013, with predictions for 2021.

In line with previous studies, incidence of MNs was markedly higher in men than in women for most subtypes. Notable exceptions included MDS associated with isolated del(5q), AML and MDS therapy related, and essential thrombocythemia, already reported in the literature. Likewise, the incidence of all MNs increased with advancing age, being particularly marked in NOS cases, in which the incidence rose sharply from age 70 years. This might suggest a decline in the quality of the diagnostic workup in the elderly, who are less likely to receive aggressive diagnostic tests due to comorbidity and/or frailty, and may therefore receive a suboptimal treatment for their conditions^23^.

Regarding specific MNs subtypes, lower incidence rates for most entities were reported in our study in comparison to the most recent data provided by the HMRN^14^. This could be partly explained by the specialized nature of the HMRN (with all diagnoses made and coded by clinical specialists working at a single integrated hematopathology laboratory), and by the lack of concordance in the recording of progressions/transformations. In contrast, incidence rates of overall MPN, MDS, and MDS/MPN in our region were markedly higher in comparison to European^2,8,13^ and US^9–11^ datasets, most of them covering years before/close to the implementation of the ICD-O-3 and the 2001 WHO classification. As far as MPN are regarded, disparities were mainly attributed to polycythemia vera and essential thrombocythemia, while rates of chronic myeloid leukemia (consistently documented since 1970’s with the identification of its causal chromosome transition), primary myelofibrosis, and mastocytosis were similar across studies. Such differences may be linked to the identification of the JAK2 mutation in 2005^24^ and the derived 2008 WHO guidelines for MPN, whose impact is not documented in series covering only previous years.

On the other hand, the incidence of AML, which is a long-established entity, was more homogeneous across different regions. Indeed, overall rates were consistent with European^2,8^ and US^25^ findings, as well as with smaller European series^13,26–28^, while slightly lower rates were reported in Canada^29^ and Switzerland^30^. Karyotypic information was not available for many of our cases, and thus, the proportion of AML with cytogenetic abnormalities (14%) was slightly lower in comparison with more specific studies^31–33^. However, rates of AML with t(15;17)(q22;q12) were still higher compared to the European average, further supporting the hypothesis that such entity might be more prevalent among individuals with Spanish ancestry^34^. Finally, most of these studies included AML of ambiguous lineage within AML-NOS subgroups, although it is placed as a distinct category from AML since the introduction of the 2008 WHO classification. Further studies are warranted to clarify the epidemiology of these entities owing their clinical relevance.

We evidenced increasing incidence trends of MDS/MPN, MDS, and several MPN, previously reported in the literature and mostly linked to refinements in the diagnostic, classification, and registration practices. Within the latter, this was particularly seen in the three most frequent Philladelphia chromosome negative subtypes, and thus, may be linked to the implementation of screening for JAK2 mutation. In the same vein, Girodon et al.^35^ documented an almost twofold increase in the incidence of essential thrombocythemia after 2005, but not in the remaining MPN subtypes. In agreement with the few European studies examining AML incidence trends^13,15^, we found a stable incidence of overall AML across the period of study. In contrast, an increasing trend was found in a Dutch pediatric study (1990–2015)^36^ and in Canada (1992–2010)^29^ and US from 2009 to 2010^25^ in the general population, the latter mainly attributed to changes in the registration of transformations in the Surveillance, Epidemiology, and End Results (SEER) program. Finally, NOS cases decreased remarkably across the period of study, which could be attributed both to a more specific clinical diagnosis and/or to an improved codification in Spanish cancer registries.

The etiology of MNs, in line with most hematological malignancies, is still uncertain. Several subtypes have been consistently associated with treatments (i.e. radiation, alkylating agents or topoisomerase II inhibitors)^37^, while environmental epidemiological studies suggest a potential role of obesity, tobacco exposure, autoimmune disorders, and infections in myelodisplastic^38^ or myeloproliferative diseases^39^. However, neither these factors, nor the genetic alterations currently described^40^, can explain the large variability in the incidence of these neoplasms^2^. In addition, drawing etiological hypothesis based on geographic heterogeneity in incidence rates is hampered by heterogeneity in accuracy and completeness in the registration of several subtypes. Several medical-claims-based studies have shown an underreporting of MNs^41–44^, namely MDS and MPN (which are often diagnosed and managed in an outpatient setting, and might be missed by surveillance systems relying on hospital registration), and among the elderly (in which diagnostic evaluation might not be as aggressively sought as in younger individuals). Indeed, we evidenced marked differences in incidence rates across Spanish provinces, with the highest incidence rates of MDS, MPN, and MDS/MPN reported in the Girona cancer registry, which has started several initiatives^15,45^ to cope with these challenges. Following the example of the French Network of Cancer Registries (FRANCIM)^46^, training programs to improve the codification and registration of hematological neoplasm have been boosted in the REDECAN during the last few years, which are expected to start to bear fruits in future studies.

Since 2008, there have been numerous advances in the identification of genetic biomarkers associated with specific MNs, which led to the release of an updated WHO classification in 2016^5^. The impact of these changes will be noticeable within the next years, when they become routinely distinguished in clinical practice and consistently coded in cancer registries. The incorporation of these updates at a cancer registry level will be eased with the release of the ICD-O-3, second revision^47^, which is recommended for use from 2020. Further studies with contemporary data including these classification changes are warranted.

The number of expected MNs in 2021 depicts the present cancer burden of these malignancies in Spain. However, these data should be interpreted with caution due to several factors. First, some subtypes are extremely rare, making estimates less robust. Furthermore, the estimates provided herein do not reflect the impact of the new 2016 WHO classification^5^, nor that of coronavirus disease 2019 (COVID-19)^48^, as they are based on extrapolations of cancer data collected in previous years. Regarding the latter, although the full extent of the impact of the COVID-19 pandemic remains unknown, delays in cancer diagnosis are expected to cause a short-term decline in cases followed by an increasing incidence of advanced-stage diagnosis^49,50^. In addition, if, over the period 2002–2013, there had been an increase in the completeness in the registration of MN cases, with the corresponding positive effect on the APC, this would cause an overestimation in the number of cases predicted for the year 2021. Nonetheless, these results are still interesting for clinicians and public health specialists in evaluating the cost of management and new treatments for these pathologies, and to account for the gap between the expected and the observed cases after the COVID-19 pandemic.

Among the strengths of this population-based study is the large number of MNs that allowed us to assess and compare incidence rates not only for common but also for relatively rare entities. However, several limitations must be considered when interpreting our results. First, the changing classification and diagnostic criteria (and consequent heterogeneity in disease definitions across countries, clinical centers, and cancer registries) hamper the interpretation of our incidence rates and trends, as well as comparisons with previous studies. In addition, we cannot exclude the aforementioned underreporting of cases, particularly documented in MDS and MPN, and among the elderly. In addition, we lacked a centralized pathology and clinical review, which could have decreased the proportion of NOS cases and improved the quality of our data. This is particularly relevant for MDS, due to the poor inter-observed concordance in diagnosis and the numerous non-neoplastic conditions that can mimic such neoplasms^51,52^. Nevertheless, in spite of the unavoidable biases due to variability and variation in registration quality and coding practices, over 95% of cases had adequate morphology specification.

In conclusion, this study presents the first comprehensive population-based analysis of MNs incidence in Spain. It highlights some useful points for public health authorities, such as the increasing incidence of several subtypes, the remarkable variability in incidence rates (especially of MDS, MPN, and MDS/MPN) among provinces, and the number of cases expected in 2021 based on these data. The negative trend in the incidence of NOS cases suggests a more specific diagnosis and/or improvements in the registration of these cases across the study period, however, additional efforts should be made to improve the quality of MNs data in future studies.

## Data Availability

The dataset analyzed during the current study is not publicly available due to national regulations of cancer registry data. However, it is available anonymized from Dr. Rafael Marcos-Gragera (rmarcos@iconcologia.net) on reasonable request.
